# The use of the social robot NAO in medical settings: how to facilitate interactions between healthcare professionals and patients with autism spectrum disorder

**DOI:** 10.3389/fpsyt.2025.1675098

**Published:** 2025-10-02

**Authors:** Federico Biagi, Cristina Iani, Luigi Biagiotti

**Affiliations:** ^1^ Department of Engineering “Enzo Ferrari”, University of Modena and Reggio Emilia, Modena, Italy; ^2^ Department of Surgery, Medicine, Dentistry and Morphological Sciences, University of Modena and Reggio Emilia, Reggio Emilia, Italy

**Keywords:** autism spectrum disorder, social robot, NAO, healthcare professionals, patients

## Abstract

**Objectives:**

This study investigates how to facilitate the use of the social robot NAO in medical settings to support interactions with children diagnosed with Autism Spectrum Disorder (ASD). The objective was to develop intuitive control methods that enable healthcare professionals to easily integrate the robot into clinical practice.

**Methods:**

Two control modes were designed: Puppet mode, where clinicians manually operate the robot via a graphical console, and Assistant mode, where a Large Language Model translates clinicians’ spoken requests into robot actions and dialogue. Twenty-three doctors evaluated both modes through video demonstrations and completed questionnaires assessing usability, usefulness, and ethical acceptability.

**Results:**

Both modes were considered effective and user-friendly. Assistant mode was perceived as more intuitive and adaptable, facilitating seamless interaction, whereas Puppet mode was judged slightly more reassuring for patients and somewhat more appropriate in terms of robot actions.

**Conclusion:**

Overall, both approaches were positively received, with Assistant mode emerging as the preferred option for integration into clinical workflows due to its perceived simplicity and flexibility. These findings highlight clinicians’ positive perceptions of two novel control modes and emphasize NAO’s potential to enhance patient engagement and reduce stress. Further empirical validation with children in real clinical trials is warranted to confirm these benefits and optimize robot-assisted interventions in ASD care.

## 1 Introduction

In the past years, progress in the robotics field has driven the emergence and diffusion of social and human-like robots. Social robots are artificial systems designed to fulfill social roles within environments where both human and non-human agents coexist (1). Their applications span diverse areas, including healthcare, medical and educational contexts, as well as entertainment scenarios. Within the healthcare sector, social robots provide cognitive and emotional support through social interaction, improving patient care and reducing patient burden, thus playing an important psychological role for the patient well-being.

Children diagnosed with ASD tend to have more frequent visits to physicians and a greater likelihood of hospitalization (2). Confronting unfamiliar environments such as medical offices and hospitals and undergoing medical tests and procedures may be particularly stressful for these children. Indeed, their difficulties in social interactions and communication, as well as the difficulties in sensory processing that may render them over-sensitive to sensory stimuli present in the environment, such as lights, noises and touch (3), may hinder the smooth completion of medical procedures, posing a burden also on healthcare professionals. Indeed, while sensory stimulation may induce discomfort and lead to high levels of stress, communication difficulties may hinder the ability to understand and follow the instructions provided by healthcare professionals. In the long term, these distressing experiences may lead to a decrease in the use of medical services. It is therefore important to implement strategies that may help overcome these challenges. One strategy is to create sensory adaptive environments, that are designed to minimize sensory-related discomfort and maximize relaxation (4, 5). Another complementary strategy is the use of social robots, which can provide engaging interactions that distract and reassure children during medical examinations, thereby contributing to a calmer and safer clinical environment.

The comprehensive analysis in (6) as well as the literature review by (7) indicate that social robots represent a promising tool to foster social skills in children and adolescents on the autism spectrum. Children with ASD often exhibit deficits in social skills and, consequently, tend to visually orient themselves toward non-social objects such as robots rather than toward human beings (8). This preference for non-social objects is commonly observed among children with ASD because robots and similar objects are perceived as more stereotyped, predictable, straightforward, and easier to understand (9). Allowing children with ASD to interact with a social robot has been shown to enhance their social skills (10) and attentional engagement (11), as well as to reduce social anxiety (12). Among the available social robots, the NAO Robot has been widely studied and shown to be effective in pediatric contexts. NAO Robots are human-like collaborative robots developed and produced by the French company Aldebaran, capable of movement and speech. Their primary purpose is to interact with young users, providing friendly and engaging interactions that can either distract or assist them during various activities (13, 14). The rationale for selecting this type of social robot is further supported by its essential characteristics:

- Appearance: simple and friendly human-like traits.
- Height: shorter than that of a child.
- Human-like locomotion: the robot moves around using bipedal walking.
- High level of autonomy: the robot can stand and move independently.
- Interaction capabilities: speech, playback of sounds and music, and movement of arms and hands.

Research studies (15–17) indicate that these features are effective in fostering an emotional connection between the robot and children with autism. Tapus et al. (18), who investigated the robot’s influence on children with autism, reported its positive impact on maintaining children’s attention during clinical sessions. As an example, research has investigated their potential in teaching specific social skills, such as joint attention, which is a fundamental deficit in children with ASD (19). A comprehensive review by Alghamdi et al. (20) analyzed 24 studies on robot-assisted interventions for children with ASD, finding that, between various employed robots, 41% of the studies used NAO robots to carry out experiments, demonstrating their widespread adoption in this field. The review reported that robot-assisted therapy showed potential for improving social interaction, communication, and emotional regulation skills in children with ASD. Beyond applications for children with ASD, in pediatric environments, the NAO Robot has proven valuable in facilitating therapeutic interventions (21). Numerous studies, including (22–24), have explored the use of the NAO Robot in the treatment and educational support of children with various disabilities, such as Attention Deficit/Hyperactivity Disorder (ADHD), language disorders, and Down syndrome.

## 2 Related works

### 2.1 Robots as companions for autistic children

The use of robots as facilitators during visits with autistic children is not new (25). In 2007, Duquette et al. (26) conducted a study using a custom mobile social robot called “Tito” to investigate the effectiveness of a robot compared to a human mediator in maintaining the attention of a group of autistic children and reducing avoidance behaviors. The authors found that children interacting with the robot exhibited more eye contact and physical proximity than those interacting with a human mediator, demonstrating the robot’s efficacy in maintaining the attention of autistic children. Moreover, the children shared facial expressions, such as smiles, with the robot, indicating the presence of empathetic responses during the interaction.

In 2012, Kim et al. (27) conducted a study involving a group of 24 autistic children interacting with both a human paired with a partner chosen from a touchscreen computer game, a social robot, or another human. The social robot used in the experiment was a dinosaur-style robot called Pleo. While playing a game specifically designed for the study, the authors highlighted that children with ASD spoke more while interacting with the social robot than with another adult. They were also eager to interact with the present adult when the robot was involved in the interaction. The robot elicited speech directed at the adult confederate, motivating improved social behavior in the children.

Similarly, in 2013, Wainer et al. (28) deployed the robot KASPAR (29) with the purpose of facilitating collaborative play with children with autism and a cooperative video game with a human involved. The study highlighted that children demonstrated more collaborative behaviors with the human after having played together with the social robot KASPAR, with some children even imitating KASPAR’s speech and actions (which did not occur with the human partner).

### 2.2 The need for intuitive control methods of the NAO robot

While the use of social robots in the medical field has been widely demonstrated, their effectiveness and benefits are closely linked to ease of use. The usability of a robotic system is a fundamental prerequisite for its successful deployment. The IEC (International Electrotechnical Commission) 62366–1 standard (30) defines usability as a characteristic of a user interface that facilitates its use, ensuring effectiveness, efficiency, and user satisfaction within the intended environment.

**Figure 1:**
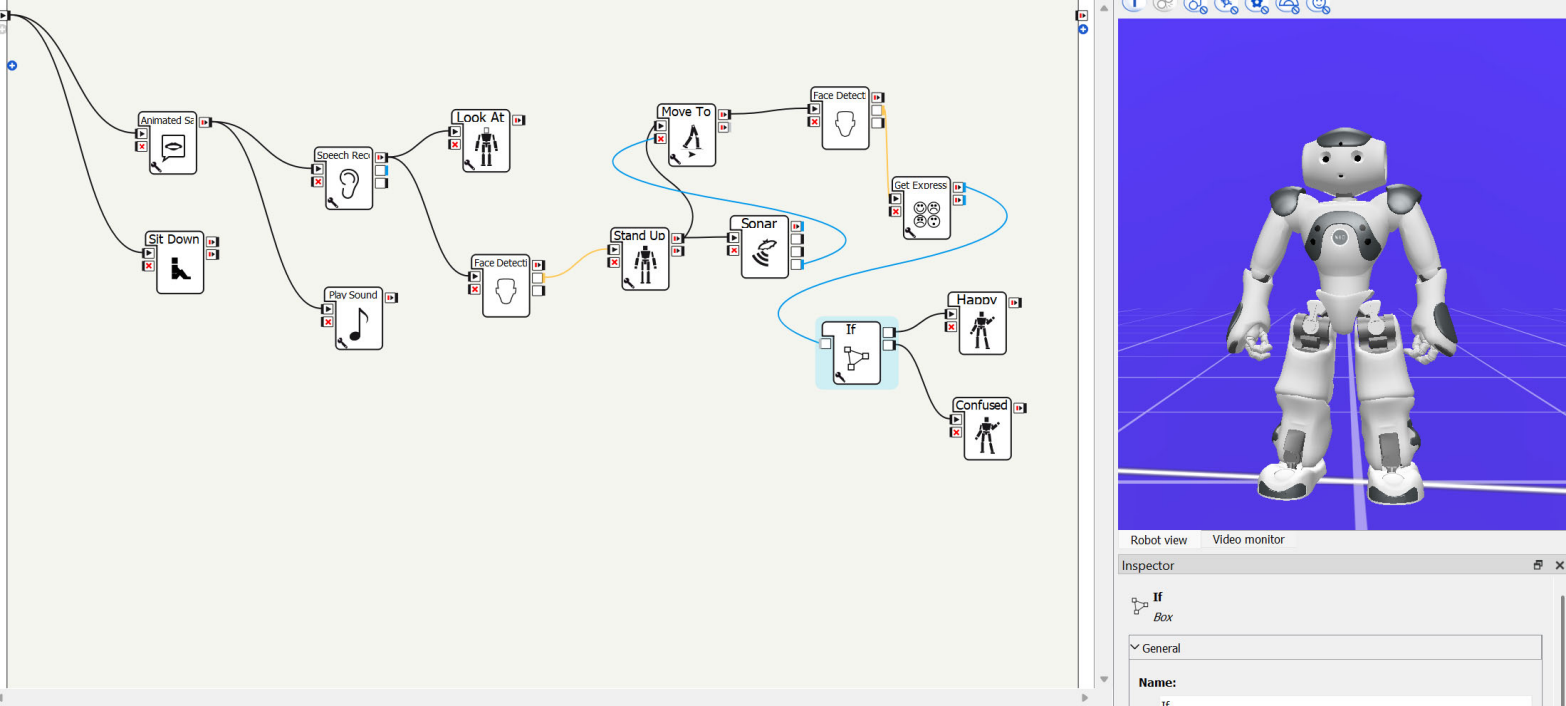
Choregraphe screen with a simple loaded program. Visual workflows can easily become confusing for complex robot routines.

Keizer et al. (31) identified the lack of a user-friendly interface as a specific limitation of the NAO robot. In their study, older adults experienced difficulties interacting with NAO through voice commands, highlighting the need for more intuitive methods of programming and control. Currently, NAO is operated via a dedicated tool called *Choregraphe*, an integrated development environment (IDE) with a graphical interface for creating robot routines. Each action is encapsulated in a block that executes a specific function, and sequences of actions can be built by connecting blocks into workflows. Although this block-based programming approach eliminates the need for manual coding, it remains challenging for non-expert users and limits its applicability, as also identified by Puglisi et al. (32). Yu et al. (33) reported that the block-linking method often leads to complex visual structures and timing issues, making the system confusing even for experienced users (**Figure 1**). Clinicians have also described the interface as unintuitive, particularly in hospital contexts where robot behaviors must be adapted rapidly during clinical visits. Such limitations hinder the effective deployment of NAO in stressful scenarios, such as medical visits with autistic children, where timely and intuitive interaction is crucial.

To overcome these challenges, several alternatives have been proposed, ranging from brain–computer interfaces for teleoperation (34) to simplified programming environments designed for children (35). Building on this line of research, the present study evaluates two novel control methods for the NAO robot. The first, *Puppet Mode*, is a console-based system that allows clinicians to manually operate the robot via a laptop. The second, *Assistant Mode*, enables NAO to act as a semi-autonomous agent that collaborates with the clinician to engage and distract the child during visits. The aim of this work is to determine which control method is preferred by clinicians, thereby establishing a practical and effective approach for integrating NAO into cardiological visits with autistic patients. To the best of our knowledge, no previous study has compared two distinct control modes for NAO with the specific goal of evaluating their usability and clinical applicability.

## 3 Materials and methods

### 3.1 Experimental setup

**Figure 2:**
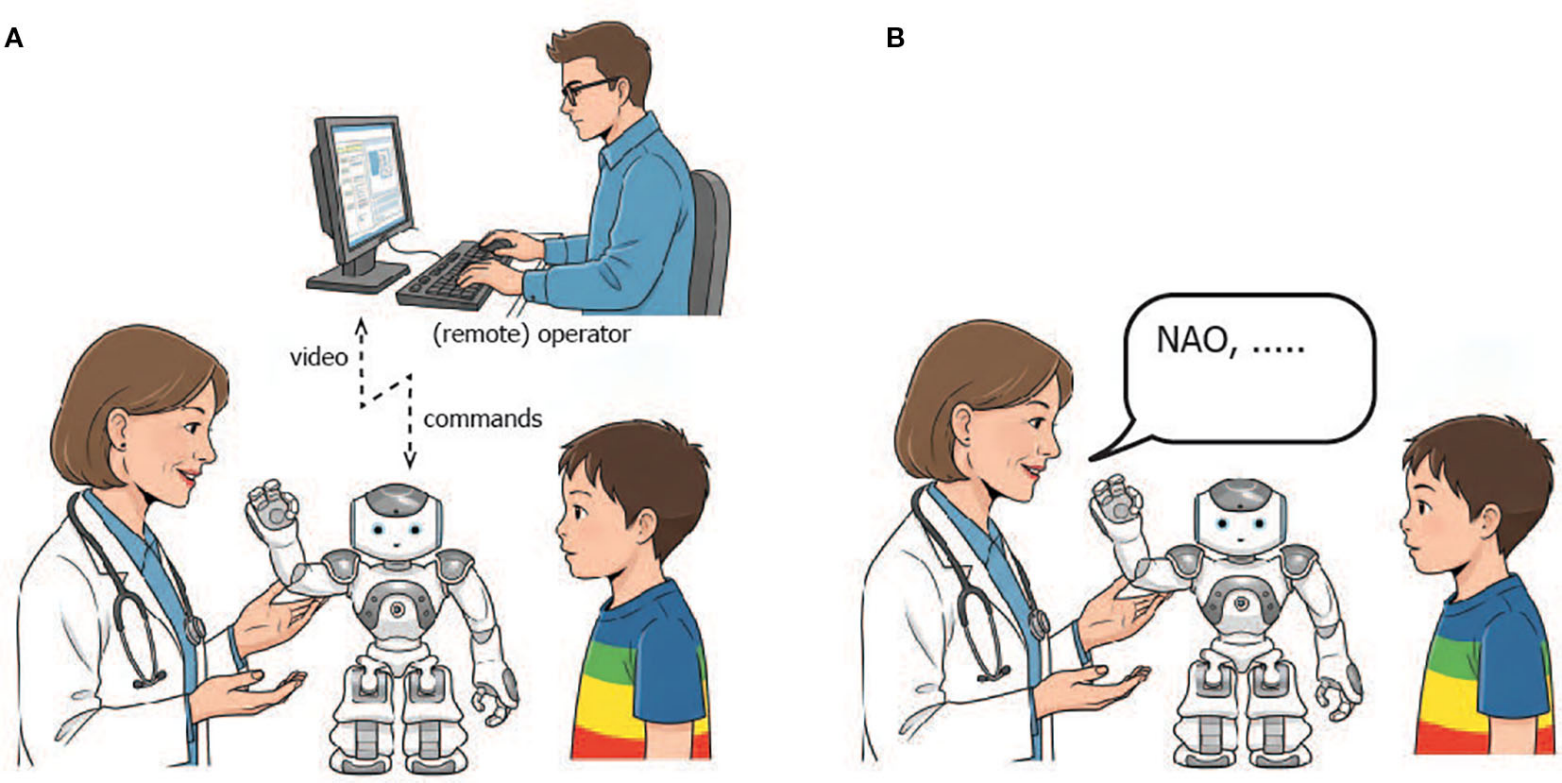
Different control modes for the NAO robot: Puppet mode **(A)** and Assistant mode **(B)**.

To identify the most effective control mode for the NAO robot from the clinicians’ perspective, we implemented two distinct control modalities, schematically illustrated in **Figure 2**. The first modality, Puppet Mode, introduces a novel console-based graphical interface specifically developed for this project. Through this interface, clinicians can directly control the movements, speech, and actions of the robot by interacting with buttons and text fields, leaving the NAO robot with no autonomy over its movements or dialogue generation. The second modality, Assistant Mode, leverages a Large Language Model (LLM) to convert the NAO robot into an autonomous Assistant capable of independently managing its limb movements and generating contextually appropriate dialogues (36). This mode grants the robot freedom in its interactions, aiming to enhance its responsiveness and adaptability during clinical encounters. To evaluate these modalities, we conducted a study involving twenty-three medical doctors.

### 3.2 Puppet mode

**Figure 3:**
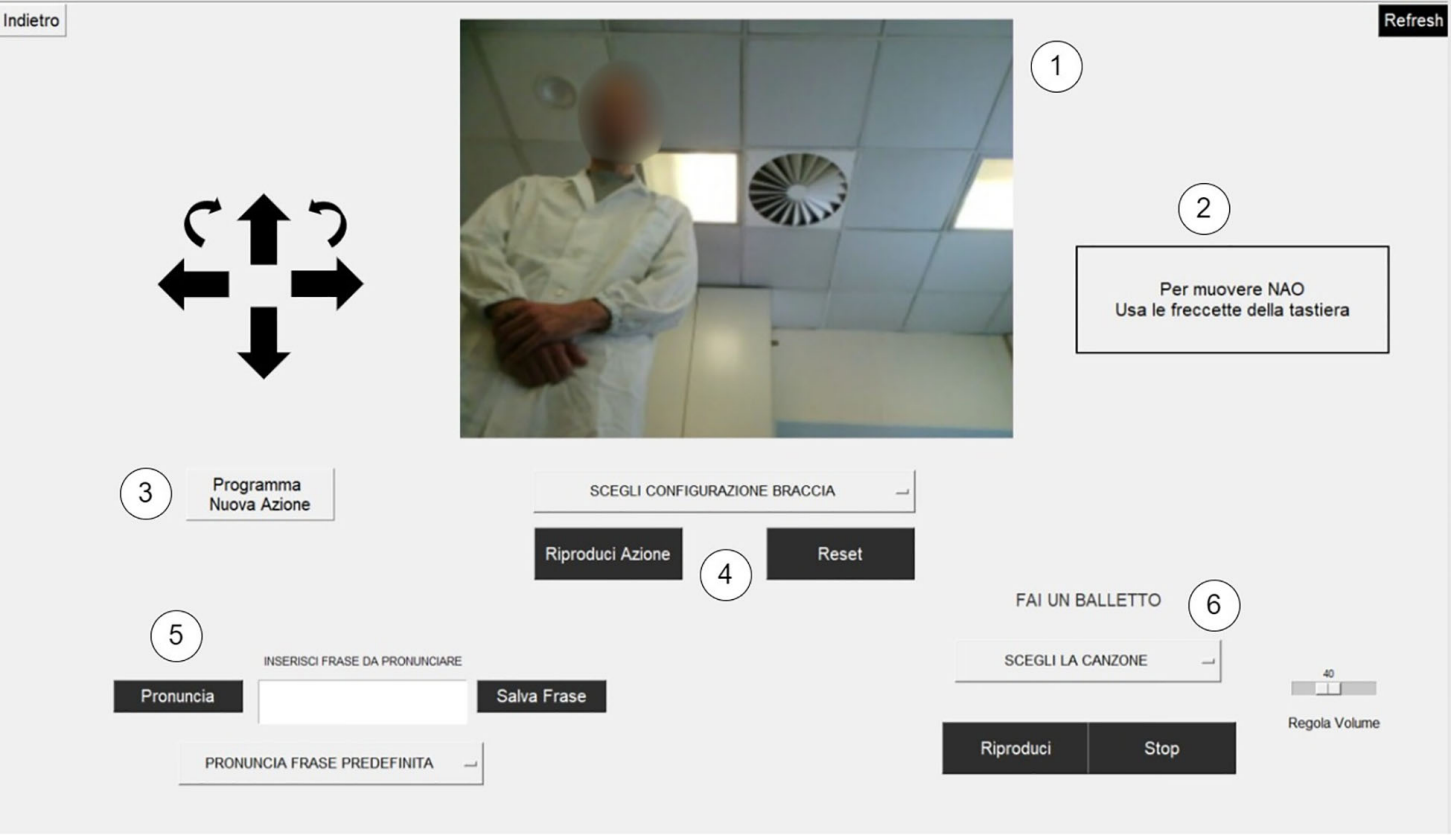
Puppet mode console screen.

The name for this modality is inspired by the term “puppet,” as the NAO robot is fully controlled by the doctor via a graphical interface. This interface is structured as a command console comprising the following components portrayed in **Figure 3**:

1. The program connects to the NAO’s head-mounted webcam and streams video data to a dedicated window, allowing the user to monitor the robot’s field of view.
2. A textual message informs the user that the NAO can be controlled using the keyboard arrow keys, enabling navigation within the environment. The robot’s head movements can also be controlled via keyboard.
3. A button opens a new screen where the user can program new movements for the NAO.
4. The doctor can select from a list of actions that the robot can perform. These actions are customizable.
5. The doctor can insert sentences in a text box. The NAO then pronounces the sentence.
6. The doctor can select from a list of dance moves that the robot can perform to entertain the patient.

The main feature of this modality is the complete control of the NAO robot via a user-friendly interface.

### 3.3 Assistant mode

**Figure 4:**
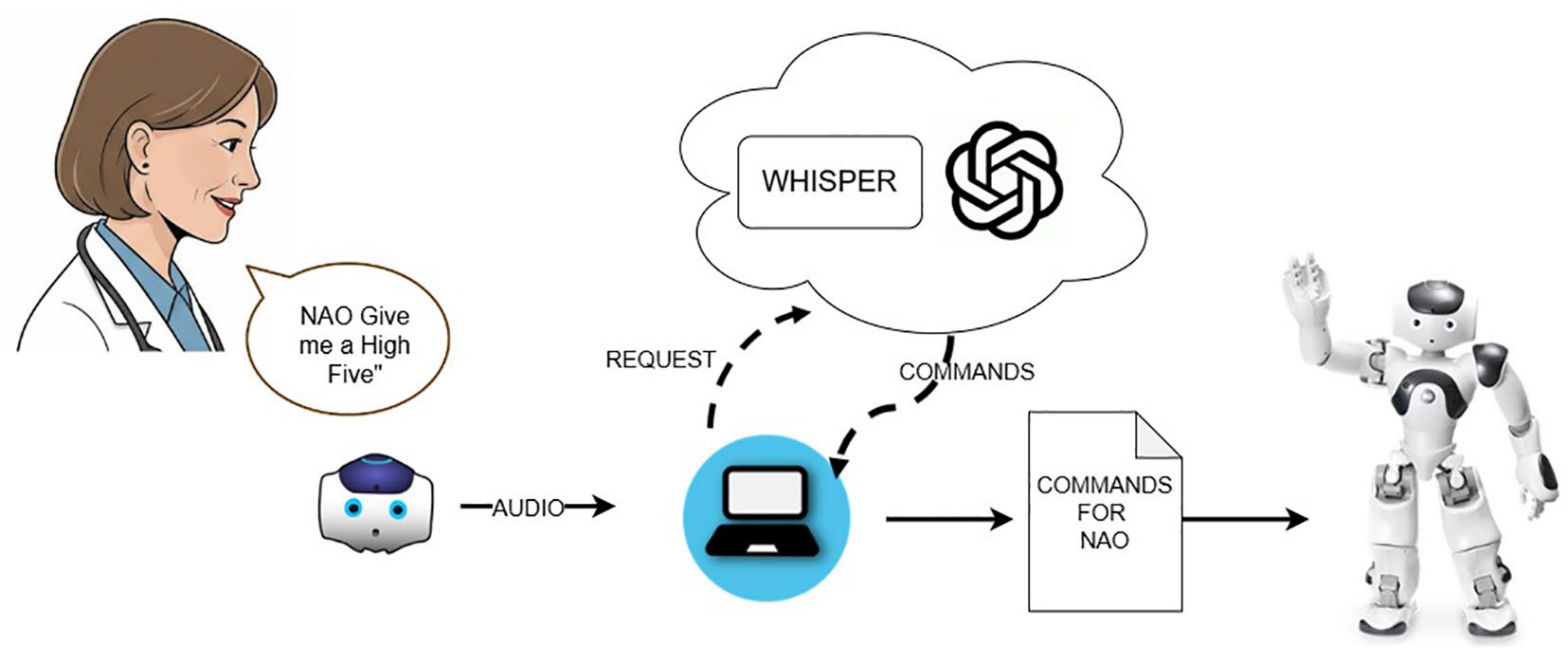
Assistant mode pipeline.

The robot becomes an autonomous assistant that cooperates with the doctor to entertain the patient. In **Figure 4**, the workflow underlying the interaction between the doctor and the NAO robot is depicted. The doctor asks the NAO to perform an action, and the audio is captured through the NAO’s microphones and transmitted to the laptop. Subsequently, the audio request is transcribed into text using the Whisper service and processed by a Large Language Model (ChatGPT was chosen for this project). The language model returns a list of actions that NAO can perform to fulfill the user’s request through actions and speech commands. The robot then executes these commands.

### 3.4 Participants

Twenty-three clinicians, all of whom were physicians with a medical degree representing a range of clinical specialties, participated in the study, which was conducted online. Of them, 30.4% attended a medical residency program at the time of the study. Demographic analysis revealed that 56.5% of the participants were female, while 43.5% were male. Regarding age distribution, 52.2% were under 35 years old, 34.8% were between 35 and 55 years, and 13.0% were over 55 years of age. Most participants were based in the province of Modena (95.6%), with the remaining 4.4% working in the Reggio Emilia province. In terms of professional experience, 52.2% of the participants reported having five years or less of work experience, 4.3% had between six and ten years, another 8.7% had between eleven and fifteen years, and 34.8% had more than fifteen years of experience. Only 17.4% of the participants reported previous experience with robots, such as the NAO (8.7%) or the Da Vinci robotic surgical system (4.3%). The study was conducted in accordance with the ethical standards laid down in the 1964 Declaration of Helsinki and its later amendments and was approved by the Ethics Committee of the University of Modena and Reggio Emilia (protocol n. 2025-UNMRCLE-0073649). Participants were recruited through a mailing list, and all gave their informed consent to participate in the study.

### 3.5 Questionnaires

Questionnaires were presented using Google Forms to assure standardized administration. A series of videos was included in the questionnaire. An introductory video explained to participants the structure and purpose of the project, providing information on its original objectives and the findings we hope to achieve. Subsequently, each section of the questionnaire was introduced by a video demonstrating a specific NAO control mode. The Puppet Mode video showed a simulated visit in which both the ambulatory setting and the graphical interface of the console were displayed on screen. In contrast, the Assistant Mode video showed only the ambulatory setting, as no console is required to control NAO in this mode. In both videos, the same type of simulated visit was performed to avoid differences that could influence the viewer’s perception. First, the patient was greeted by the NAO robot and introduced to a cardiological visit. Next, the robot asked the patient to relax and sit on a bed while explaining the steps of the visit. Subsequently, NAO entertained children with dances and fairy tales. Finally, NAO congratulated the patient and says goodbye.

To assess participants’ ethical acceptability of social robots used with children diagnosed with ASD we employed the Ethical Acceptability Scale developed by Peca et al. (37) (see **Appendix Table 1**). The scale comprises 12 statements (items): five items assess ethical acceptability for use, four items assess ethical acceptability of human-like interaction, and three items assess ethical acceptability of non-human appearance. Participants were asked to indicate their level of agreement with each statement using a five-point Likert scale where 1 corresponds to “strongly disagree”, 2 to “disagree”, 3 to “neither agree nor disagree” (neutral), 4 to “agree”, and 5 to “strongly agree”. For the present study, the original English version of the scale was translated into Italian by one person fluent in English and subsequently back translated into English by another person to assure accuracy of the translation. To assess participants’ perceptions, attitudes, perceived ease of use, and perceived usefulness of the two NAO control modes, we developed an *ad-hoc* questionnaire consisting of 17 statements (see **Appendix Table 2**). The questionnaire was developed drawing from established questionnaires assessing usability such as the System Usability Scale (38). Participants were asked to indicate their level of agreement with each statement using a five-point Likert scale where 1 corresponds to “strongly disagree”, 2 to “disagree”, 3 to “neither agree nor disagree” (neutral), 4 to “agree”, and 5 to “strongly agree”. Finally, participants were asked to express their preference for one of the proposed modalities for three different contexts (**Appendix Table 3**).

### 3.6 Procedure

Participants were first provided with a general description of the study, along with a consent form. Upon agreeing to participate, they were asked to complete a series of demographic questions regarding their gender, age range, professional position, medical specialty, work location, and prior experience with robots. These questions were followed by the administration of the Ethical Acceptability Scale (37). Subsequently, participants viewed the first video, which depicted the simulation of an interaction between a doctor and the NAO robot. Upon completion of the video, participants were asked to complete an *ad hoc* questionnaire. After a brief pause, participants were shown a second video, followed by the same questionnaire used after the first video (but rephrased to address the specific control mode). The two videos demonstrated the two different control modes: Puppet and Assistant. To control for potential order effects, the sequence in which the videos were presented was counterbalanced: 10 participants viewed the video of the Puppet control mode first, while 13 viewed the video of the Assistant control mode first. At the end of the study, participants were asked to indicate which mode they considered the easiest to use, which mode they found most useful in clinical practice, and which mode they would be most willing to adopt during clinical practice.

## 4 Results

All statistical analyses were performed using IBM SPSS Statistical software (version 25.0) and JASP (version 0.19.1; Jasp Team, 2024). Given the nature of the data, nonparametric tests were used: the Wilcoxon test was used to assess whether scores for the EAS and post-video questionnaire were significantly different from the neutral value of 3. For the post-video questionnaires only, the Wilcoxon signed-rank test was used to assess score differences between control modes. For the three questions administered at the end of the study, the chi-square test was used to assess whether the observed frequencies were significantly different from what was expected (that is, equal frequencies for the two control modes). For all statistical tests the alpha level was set to 0.05.

### 4.1 EAS

**Figure 5:**
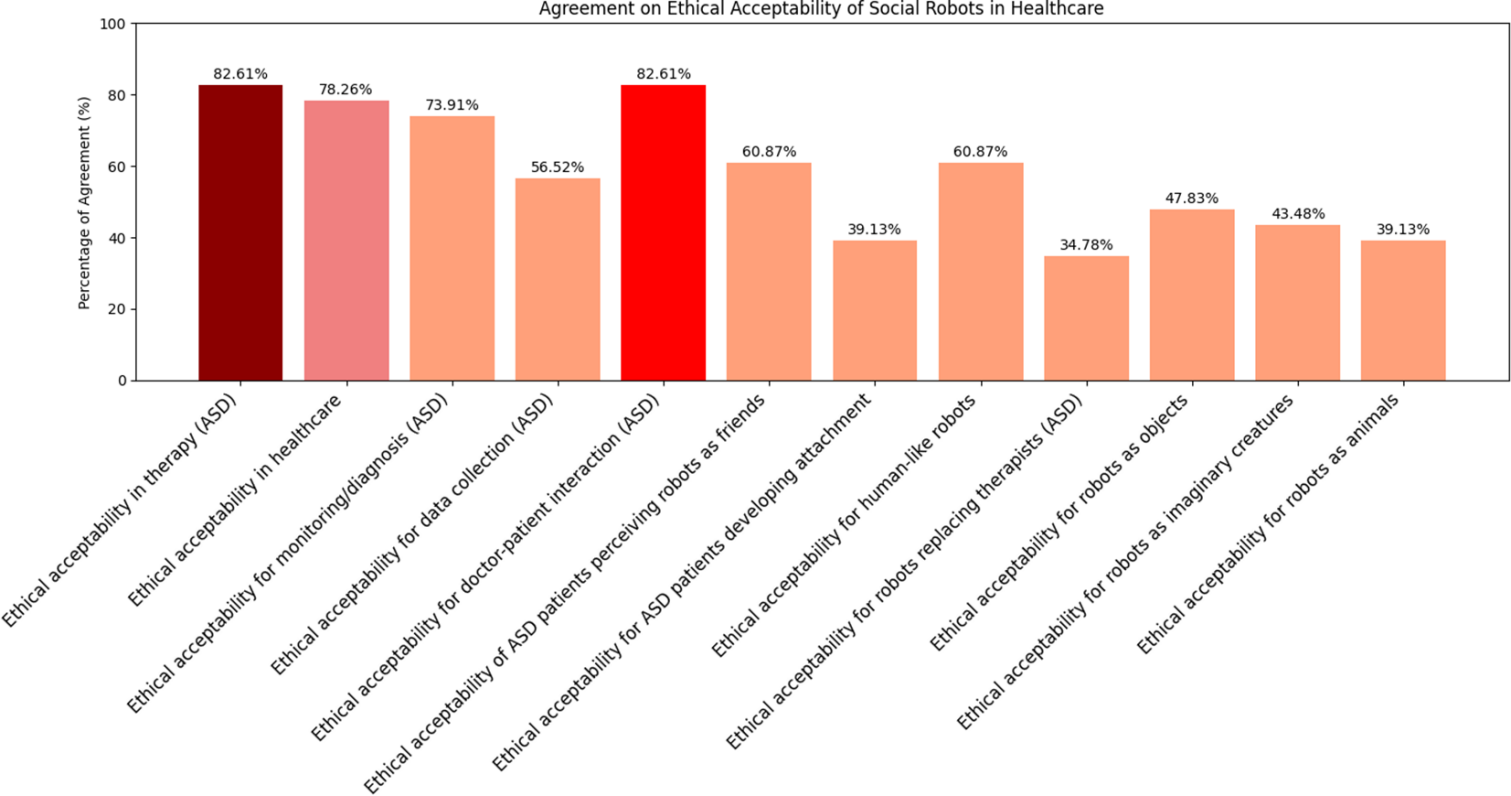
Agreement percentages for each statement in the EAS questionnaire. The three statements with the highest levels of agreement are highlighted using darker colors in the bar plots, distinguishing them from the others.

**Figure 6:**
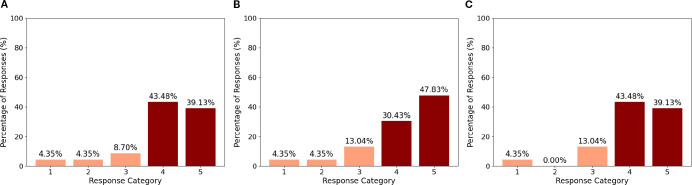
Ethical acceptability of the robot in therapy involving ASD **(A)**, in healthcare **(B)**, in doctor-patient interaction **(C)**.

Mean scores, standard deviations, and Wilcoxon test results for the 12 items of the Ethical Acceptance Scale are reported in **Appendix Table 4**. Agreement was considered when the response was *>* 3. All scores were significantly greater than 3 except for the following items, whose scores did not significantly differ from 3 (ps *>* = 0.25): “It is ethically acceptable that children become attached to social robots” (M = 3.17, SD = 1.15), “It is ethically acceptable to use social robots that replace therapists for teaching social skills to children with autism” (M = 2.91, SD = 1.38), “It is ethically acceptable to make social robots that look like imaginary creatures” (M = 3.30, SD = 1.19), and “It is ethically acceptable to make social robots that look like animals” (M = 3.17, SD = 1.19). The analysis of the distribution of the responses to the items belonging to the ethical acceptability of use sub-scale (**Figure 5**) showed that most of the respondents agrees with using robots in healthcare, as indicated by the 78.2% of agreement with the item “It is ethically acceptable that social robots are used in healthcare” (**Figure 6B**), and specifically with ASD children, as indicated by the 82.6% of agreement with the item “It is ethically acceptable that social robots are used in therapy for children with autism” (**Figure 6A**). The item “It is ethically acceptable that social robots are used in therapy to support the interaction between the therapist and the child with autism” obtained 82.6% of agreement (**Figure 6C**), indicating that social robots are perceived as possible mediators between therapists and children.

### 4.2 Post-video questionnaire

Mean scores, standard deviations, and Wilcoxon test results for the post-video questionnaire, as a function of control mode, are reported in **Appendix Table 5**. The distribution of responses to the questionnaire items for the two control modes is presented in **Appendix Table 2**. Agreement with each statement was calculated as the percentage of participants selecting option 4 (“agree”) or 5 (“strongly agree”) on the Likert scale. Neither control mode was perceived as complex to use, as indicated by the low agreement with the statements “I found the robot NAO complex to use in the Assistant modality” (M = 2.43, SD = 1.16; 17.4% agreement), and “I found the robot NAO complex to use in the Puppet modality” (M = 2.52, SD = 0.99; 17.4% agreement). Similarly, neither mode was considered as frustrating, as indicated by the low agreement with the statements “Using the NAO robot in Assistant mode is frustrating” (M = 2.22, SD = 1.04; 13.0% agreement) and “Using the NAO robot in Puppet mode is frustrating” (M = 2.09, SD = 0.90; 4.4% agreement). Although agreement with the statement “The robot responds quickly enough” was low (M = 2.96, SD = 1.02; 8.7% agreement for the Assistant mode; M = 3.44, SD = 0.79; 8.7% agreement for the Puppet mode), the actions performed by the robot were considered appropriate with respect to the predetermined objectives (M = 3.48, SD = 1.08, 52.2% agreement for the Assistant mode; M = 3.70, SD = 0.63, 69.6% agreement for the Puppet mode). Furthermore, the interaction mode was judged as sufficiently rich in relation to the needs (M = 3.40, SD = 0.84, 43.5% agreement for the Assistant mode; M = 3.48, SD = 0.80, 56.5% agreement for the Puppet mode). Importantly, participants agreed with the statements “I think the use of the NAO robot in Assistant mode may reassure the patients” (M = 3.52, SD = 1.04; 52.2% agreement) and “I think the use of the NAO robot in Puppet mode may reassure the patients” (M = 3.61, SD = 1.16; 69.6% agreement). Agreement with the statement “I would like to use the NAO robot in Assistant mode during medical examinations” was 43.5% (M = 3.43, SD = 1.24), while it was 52.2% for the statement “I would like to use the NAO robot in Puppet mode during medical examination” (M = 3.65, SD = 1.07). This suggests that participants were slightly keener to using the NAO robot in Puppet mode compared to Assistant mode during medical examinations. Importantly, a substantial majority agreed that most people can learn to use the NAO robot in both the Assistant (M = 4.00, SD = 0.05, 73.9% agreement) and Puppet (M = 3.74, SD = 0.75, 65.2% agreement) mode. This result indicates a generally positive perception regarding the accessibility and ease of learning to operate the NAO robot, regardless of the mode. No significant differences were observed between the two control modes in the post-video questionnaire (all ps *>* 0.11), except for the statement “I think I need the support of someone who is already able to use the NAO robot”. For this item, participants reported a significantly higher level of agreement in the Puppet condition (43.5%, M = 3.22, SD = 1.09) compared to the Assistant condition (26.1%, M = 2.53, SD = 1.08), critical z-value=-2.471, p =.011. The post-study questions showed a slight preference for the Assistant mode which was indicated by 69.9% of the participants as the easiest to use (*χ* ^2^ = 3.522, p =.06) and as the modality they would be most willing to adopt during clinical practice (*χ* ^2^ = 3.522, p =.06). However, no difference emerged when participants judged the usefulness of the two 278 modes: 56.5% of the participants considered the Assistant mode to be the most useful in clinical practice, whereas 43.5% preferred the Puppet mode (*χ* ^2^ =0.391, p =.53).

## 5 Discussion

**Figure 7:**
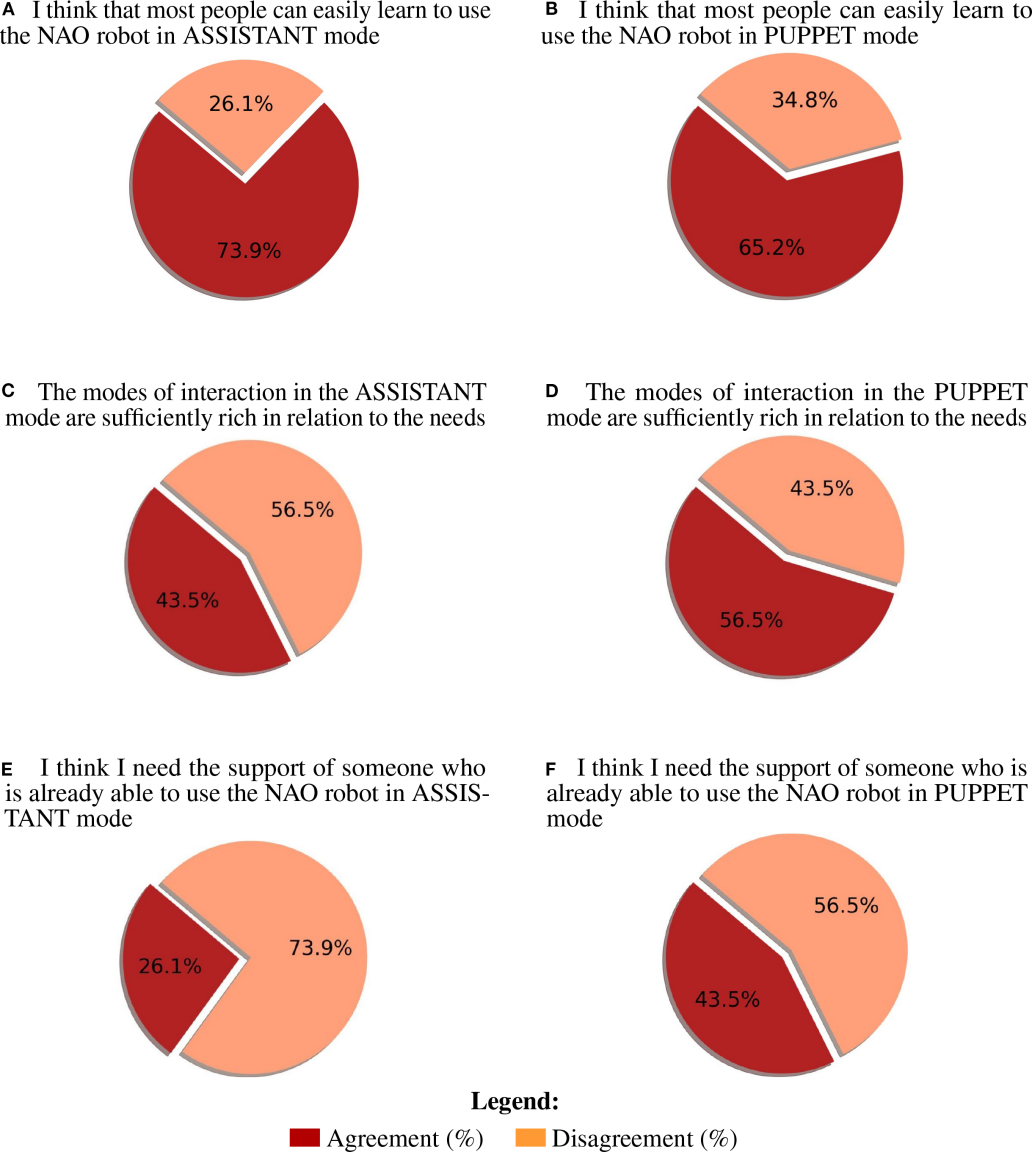
Most impactful agreement (vote 4-5) and disagreement (vote 1-3) percentages for post-video questionnaire. **(A)** I think that most people can easily learn to use the NAO robot in ASSISTANT mode **(B)** I think that most people can easily learn to use the NAO robot in PUPPET mode **(C)** The modes of interaction in the ASSISTANT mode are sufficiently rich in relation to the needs **(D)** The modes of interaction in the PUPPET mode are sufficiently rich in relation to the needs **(E)** I think I need the support of someone who is already able to use the NAO robot in ASSISTANT mode **(F)** I think I need the support of someone who is already able to use the NAO robot in PUPPET mode.

**Figure 8:**
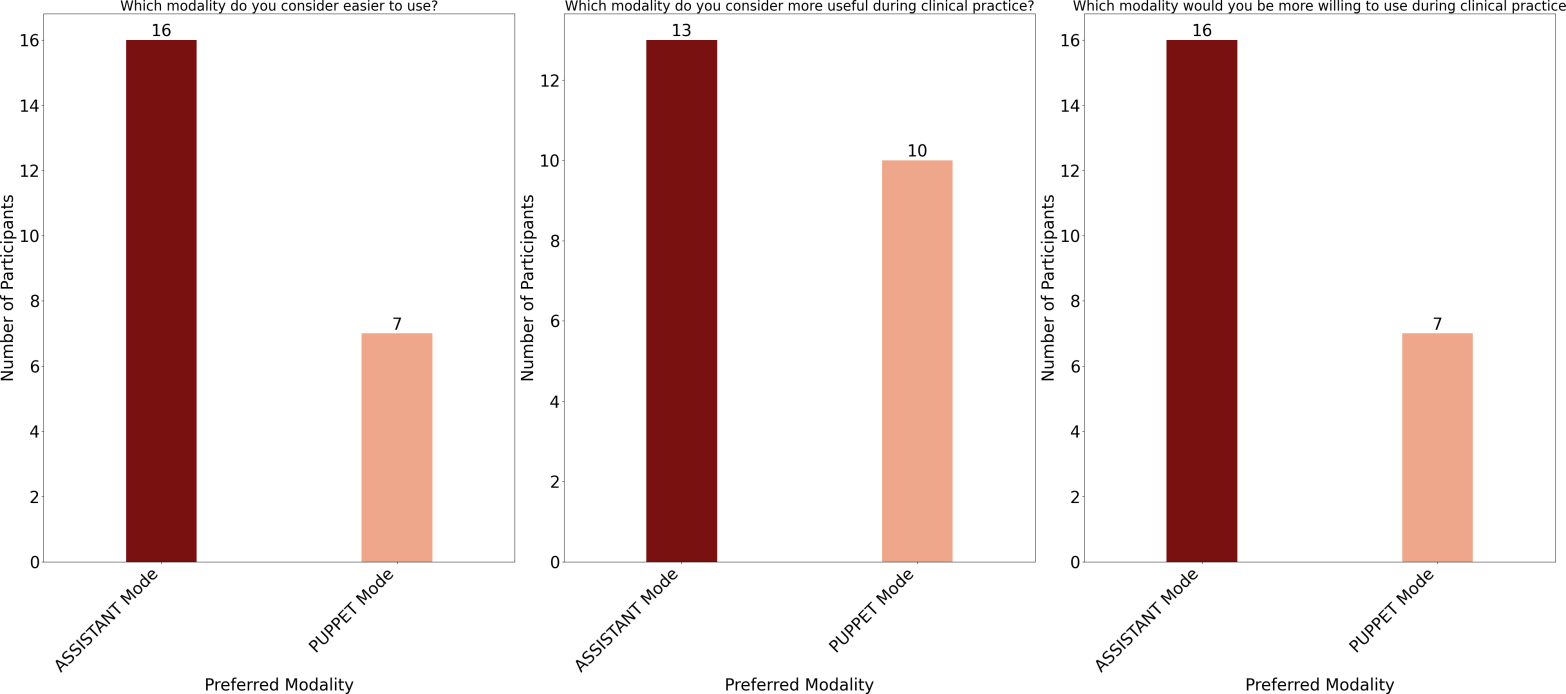
Final evaluation of the preferred control modality. The results highlight the ASSISTANT mode as the preferred modality.

This study aims to facilitate the integration of the NAO robot into clinical practice by evaluating and comparing two different control modes: the AI-driven Assistant mode and the manually controlled Puppet mode. The objective is to identify the most effective and user-friendly approach for the medical staff to adopt. To pursue this goal, we designed a series of questionnaires with video demonstrations to directly capture and interpret the clinicians’ needs and opinions. The results of the Ethical Acceptability Scale clearly showed that a significant majority of the respondents agreed with using robots in the healthcare context in general and with ADS children in specific. Such a result is in agreement with what found by (37) and is indicative of a positive attitude towards the use of social robots in healthcare. The results of the post-video questionnaires provide important indication on how the two NAO control modes were perceived. Overall, both control modes were viewed positively in terms of usability and learnability, with minor differences in preference and perceived need for support (**Figure 7**). The Assistant mode was slightly favored for ease of use and willingness to adopt, while the Puppet mode was seen as more reassuring for patients and somewhat more appropriate in terms of robot actions. Neither the Assistant nor the Puppet control mode was perceived as complex or frustrating to use. Agreement with statements regarding complexity and frustration was low for both modes, as reflected by low mean scores and low percentages of participants selecting “agree” or “strongly agree.” Participants generally did not agree that the robot responded quickly enough in either mode; however, they considered the robot’s actions appropriate for the predetermined objectives, with higher agreement for the Puppet mode. Both modes were judged as sufficiently rich to meet the needs of the interaction, with slightly higher agreement for the Puppet mode. Participants believed that the use of the NAO robot in both modes could help reassure patients, with a higher percentage agreeing for the Puppet mode. A larger proportion of participants expressed willingness to use the NAO robot in Puppet mode during medical examinations compared to Assistant mode, though the difference was modest. The majority of the participants agreed that most people could learn to use the NAO robot in both modes, indicating a positive perception of its accessibility and ease of learning. No significant differences were found between the two control modes on most questionnaire items (all p-values *>* 0.11), except for the statement regarding the need for support from someone experienced with the NAO robot. Here, participants in the Puppet mode reported a significantly higher need for support than those in the Assistant mode (p = .011). Finally, a slight majority indicated a preference for the Assistant mode as the easiest to use and as the modality they would most likely adopt in clinical practice; however, these differences did not reach conventional levels of statistical significance (p = .06) and should therefore be interpreted only as descriptive trends. No significant difference was found regarding which mode was considered most useful in clinical practice (**Figure 8**). The results of the final preference questionnaire indicate that the participants slightly preferred the Assistant mode in terms of ease of use and willingness to adopt it in clinical practice. Although participants slightly favored the Assistant mode descriptively, these differences were not statistically significant (p = .06 for adoption; p = .53 for usefulness). As such, these findings should be considered as indicative tendencies rather than firm evidence. Any interpretation must remain cautious, though the descriptive preference may still reflect clinicians’ inclination toward simplicity and intuitive interaction. This preference can be understood considering the practical realities of social robots in clinical environments, where healthcare professionals often prioritize simplicity, intuitive interfaces, and interaction richness. The Assistant mode, which requires less active and continuous control compared to the Puppet mode, may align more closely with clinicians’ needs for intuitive interaction. This could also translate in reduced operational demands during examinations, although this conclusion must be qualified by the fact that participants evaluated video demonstrations rather than direct hands-on interactions. Furthermore, while the Puppet mode was perceived as more reassuring for patients and offered a higher perceived appropriateness in robot actions, the additional control it requires may translate into a need for more training and support (as reflected in the significantly higher reported need for support in the Puppet mode). This may discourage the application of NAO robot where time and resources for staff training are limited. Thus, the preference for the Assistant mode likely reflects a trade-off between maintaining patient comfort and ensuring ease of integration into routine clinical practice.

While promising, the results of this study reflect only clinicians’ perceptions based on video demonstrations of the two control modes. Future research should involve doctors in real-life trial sessions and clinical applications with children with ASD to better assess preferences between the two approaches. In addition, studies that directly investigate patients’ perceptions of the control modes could provide stronger evidence regarding the potential benefits of implementing the NAO robot in healthcare. The work was conducted with a limited group of physicians drawn from a specific geographical setting. While this provided focused insights into clinicians’ perspectives, it may restrict the extent to which the results can be generalized across different professional roles and healthcare contexts. To extend the findings, future investigations should involve a broader range of medical disciplines. This would allow a more comprehensive assessment of the NAO robot’s applicability and could reveal clinical domains in which its introduction would be advantageous. Finally, longitudinal studies are needed to examine how sustained use of the NAO robot in both control modes influences acceptance, training requirements, and integration into clinical workflows over time.

## Data Availability

The datasets presented in this study can be found in online repositories. The names of the repository/repositories and accession number(s) can be found below: https://github.com/federicobiagi/NAO_ASD_repository.
