# Supplementary material for: The use of the social robot NAO in medical settings: how to facilitate interactions between healthcare professionals and patients with autism spectrum disorder
Source: Front Psychiatry. 2025 Oct 2;16:1675098. doi: 10.3389/fpsyt.2025.1675098 (PMC12528083; doi:10.3389/fpsyt.2025.1675098)
Supplement: Supplementary file 1 [file DataSheet1.pdf]

APPENDIX

Ethical Acceptability Scale

Table 1. Ethical Acceptability Questionnaire

Response Scale Legend

Scale	Meaning
1	Strongly Disagree
2	Disagree
3	Neither Agree nor Disagree
4	Agree
5	Strongly Agree

Sub-scale: Ethical acceptability for use

1. It is ethically acceptable that social robots are used in therapy for children with autism.
2. It is ethically acceptable that social robots are used in healthcare.
3. It is ethically acceptable that social robots are used to monitor the progress and help in the diagnosis process of a child with autism.
4. It is ethically acceptable that information is recorded and stored by a robot when it interacts with a child with autism.
5. It is ethically acceptable that social robots are used in therapy to support the interaction between the therapist and the child with autism.

Sub-scale: Ethical acceptability of human-like interaction

6. It is ethically acceptable that, as a result of their therapy, children with autism perceive social robots as friends.
7. It is ethically acceptable that children become attached to social robots.
8. It is ethically acceptable to make social robots that look like humans.
9. It is ethically acceptable to use social robots that replace therapists for teaching skills to children with autism.

Sub-scale: Ethical acceptability of non-human appearance

10. It is ethically acceptable to make social robots that look like objects.
11. It is ethically acceptable to make social robots that look like imaginary creatures.
12. It is ethically acceptable to make social robots that look like animals.

Post-video Questionnaire

1. I found the robot NAO complex to use in the ASSISTANT/PUPPET mode
2. The robot responds quickly enough (ASSISTANT/PUPPET)
3. The actions performed by the robot are appropriate with respect to the predetermined objectives (ASSISTANT/PUPPET)
4. The modes of interaction are sufficiently rich in relation to the needs (ASSISTANT/PUPPET)

5. I would like to use the NAO robot in ASSISTANT/PUPPET mode during medical examinations
6. It seems to me that the NAO robot interferes with the way I prefer to organize my work (ASSISTANT/PUPPET)
7. I felt comfortable using the NAO Robot in ASSISTANT/PUPPET mode
8. I think that most people can easily learn to use the NAO robot in ASSISTANT/PUPPET mode
9. I think the use of the NAO robot in ASSISTANT/PUPPET mode during clinical practice can increase efficiency
10. I think the use of the NAO robot in ASSISTANT/PUPPET mode during clinical practice can make the work easier
11. I think the use of the NAO robot in ASSISTANT/PUPPET mode during clinical practice can speed up certain procedures
12. I think the use of the NAO robot in ASSISTANT/PUPPET mode may be useful only with certain types of patients
13. I think the use of the NAO robot in ASSISTANT/PUPPET mode may frighten the patients
14. I think the use of the NAO robot in ASSISTANT/PUPPET mode may reassure the patients
15. I think I need the support of someone who is already able to use the NAO robot (ASSISTANT/PUPPET)
16. I can understand what to do based solely on the information provided by the system (ASSISTANT/PUPPET)
17. Using the NAO robot in ASSISTANT/PUPPET mode is frustrating

Response Scale Legend

Scale	Meaning
1	Strongly Disagree
2	Disagree
3	Neither Agree nor Disagree
4	Agree
5	Strongly Agree

Table 2 NAO Robot User Experience - Frequency Analysis

Questionnaire Entry	Response Scale (Frequency/Percentage)				
	1	2	3	4	5
**COMPLEXITY OF USE**					
I found NAO complex to use (ASSISTANT)	6 (26.1%)	6 (26.1%)	7 (30.4%)	3 (13.0%)	1 (4.3%)
I found NAO complex to use (PUPPET)	4 (17.4%)	7 (30.4%)	8 (34.8%)	4 (17.4%)	0 (0.0%)
**RESPONSE TIME**					
NAO responds quickly enough (ASSISTANT)	1 (4.3%)	7 (30.4%)	9 (39.1%)	4 (17.4%)	2 (8.7%)
NAO responds quickly enough (PUPPET)	0 (0.0%)	2 (8.7%)	11 (47.8%)	8 (34.8%)	2 (8.7%)
**APPROPRIATENESS OF ACTIONS**					
Actions are appropriate with respect to predetermined objectives (ASSISTANT)	1 (4.3%)	3 (13.0%)	7 (30.4%)	8 (34.8%)	4 (17.4%)
Actions are appropriate with respect to predetermined objectives (PUPPET)	0 (0.0%)	1 (4.3%)	6 (26.1%)	15 (65.2%)	1 (4.3%)
**INTERACTION RICHNESS**					
Interaction modes are sufficiently rich (ASSISTANT)	0 (0.0%)	3 (13.0%)	10 (43.5%)	8 (34.8%)	2 (8.7%)
Interaction modes are sufficiently rich (PUPPET)	0 (0.0%)	3 (13.0%)	7 (30.4%)	12 (52.2%)	1 (4.3%)
**WILLINGNESS TO USE**					
I would like to use NAO during medical examinations (ASSISTANT)	2 (8.7%)	2 (8.7%)	9 (39.1%)	4 (17.4%)	6 (26.1%)
I would like to use NAO during medical examinations (PUPPET)	1 (4.3%)	1 (4.3%)	9 (39.1%)	6 (26.1%)	6 (26.1%)
**WORK INTERFERENCE**					
NAO interferes with work organization (ASSISTANT)	4 (17.4%)	5 (21.7%)	9 (39.1%)	2 (8.7%)	3 (13.0%)

NAO interferes with work organization (PUPPET)	3 (13.0%)	7 (30.4%)	8 (34.8%)	2 (8.7%)	3 (13.0%)
**COMFORT OF USE**					
I felt comfortable using NAO (ASSISTANT)	1 (4.3%)	2 (8.7%)	9 (39.1%)	8 (34.8%)	3 (13.0%)
I felt comfortable using NAO (PUPPET)	1 (4.3%)	2 (8.7%)	8 (34.8%)	10 (43.5%)	2 (8.7%)
**EASE OF LEARNING**					
Most people can easily learn to use NAO (ASSISTANT)	0 (0.0%)	1 (4.3%)	5 (21.7%)	10 (43.5%)	7 (30.4%)
Most people can easily learn to use NAO (PUPPET)	0 (0.0%)	1 (4.3%)	7 (30.4%)	12 (52.2%)	3 (13.0%)
**EFFICIENCY**					
NAO can increase efficiency during clinical practice (ASSISTANT)	1 (4.3%)	3 (13.0%)	10 (43.5%)	7 (30.4%)	2 (8.7%)
NAO can increase efficiency during clinical practice (PUPPET)	1 (4.3%)	2 (8.7%)	9 (39.1%)	10 (43.5%)	1 (4.3%)
**WORK SIMPLIFICATION**					
NAO can make work easier during clinical practice (ASSISTANT)	1 (4.3%)	3 (13.0%)	9 (39.1%)	9 (39.1%)	1 (4.3%)
NAO can make work easier during clinical practice (PUPPET)	1 (4.3%)	1 (4.3%)	10 (43.5%)	8 (34.8%)	3 (13.0%)
**EXAMINATION DURATION**					
NAO can speed up certain procedures (ASSISTANT)	0 (0.0%)	2 (8.7%)	12 (52.2%)	8 (34.8%)	1 (4.3%)
NAO can speed up certain procedures (PUPPET)	0 (0.0%)	5 (21.7%)	10 (43.5%)	5 (21.7%)	3 (13.0%)
**PATIENT SPECIFICITY**					
NAO is useful only with certain patient types (ASSISTANT)	0 (0.0%)	3 (13.0%)	8 (34.8%)	7 (30.4%)	5 (21.7%)
NAO is useful only with certain patient types (PUPPET)	0 (0.0%)	3 (13.0%)	11 (47.8%)	5 (21.7%)	4 (17.4%)
**PATIENT FEAR**					
NAO may frighten patients (ASSISTANT)	0 (0.0%)	11 (47.8%)	7 (30.4%)	4 (17.4%)	1 (4.3%)
NAO may frighten patients (PUPPET)	3 (13.0%)	8 (34.8%)	9 (39.1%)	1 (4.3%)	2 (8.7%)
**PATIENT REASSURANCE**					
NAO may reassure patients (ASSISTANT)	1 (4.3%)	2 (8.7%)	8 (34.8%)	8 (34.8%)	4 (17.4%)

NAO may reassure patients (PUPPET)	2 (8.7%)	2 (8.7%)	3 (13.0%)	12 (52.2%)	4 (17.4%)
**NEED FOR SUPPORT**					
I need the support of someone who is already able to use NAO (ASSISTANT)	4 (17.4%)	9 (39.1%)	4 (17.4%)	6 (26.1%)	0 (0.0%)
I need the support of someone who is already able to use NAO (PUPPET)	2 (8.7%)	3 (13.0%)	8 (34.8%)	8 (34.8%)	2 (8.7%)
**SYSTEM CLARITY**					
I can understand what to do based solely on the information provided by the system (ASSISTANT)	2 (8.7%)	4 (17.4%)	11 (47.8%)	5 (21.7%)	1 (4.3%)
I can understand what to do based solely on the information provided by the system (PUPPET)	1 (4.3%)	3 (13.0%)	12 (52.2%)	5 (21.7%)	2 (8.7%)
**FRUSTRATION LEVEL**					
Using NAO is frustrating (ASSISTANT)	7 (30.4%)	7 (30.4%)	6 (26.1%)	3 (13.0%)	0 (0.0%)
Using NAO is frustrating (PUPPET)	7 (30.4%)	8 (34.8%)	7 (30.4%)	1 (4.4%)	0 (0.0%)

Table 3. Final Control Mode Preference

Question	ASSISTANT	PUPPET
Which modality do you consider easier to use?	16	7
Which modality do you consider more useful during clinical practice?	13	10
Which modality would you be more willing to use during clinical practice	16	7

Note. Values represent the number of responses for each modality.

Table 4. Mean scores, with standard deviations, and results of the Wilcoxon signed-ranked test for the Ethical Acceptability Scale (EAS)

Items	N	Mean	SD	V	p-value
It is ethically acceptable that social robots are used in therapy for children with autism	23	4.087	1.041	208.5	<0.001*
It is ethically acceptable that social robots are used in healthcare	23	4.130	1.100	191.0	<0.001*
It is ethically acceptable that social robots are used to monitor the progress and help in the diagnosis process of a child with autism	23	3.957	1.022	190.5	<0.001*
It is ethically acceptable that information is recorded and stored by a robot when it interacts with a child with autism	23	3.609	0.941	116.5	0.004*
It is ethically acceptable that social robots are used in therapy to support the interaction between the therapist and the child with autism	23	4.130	0.968	194.5	<0.001*
It is ethically acceptable that, as a result of their therapy, children with autism perceive social robots as friends	23	3.783	1.085	119.0	0.004*
It is ethically acceptable that children become attached to social robots	23	3.174	1.154	72.0	0.249
It is ethically acceptable to make social robots that look like humans	23	3.652	0.832	122.0	0.002*
It is ethically acceptable to use social robots that replace therapists for teaching skills to children with autism	23	2.913	1.379	80.00	0.606
It is ethically acceptable to make social robots that look like objects	23	3.522	0.947	88.5	0.010*
It is ethically acceptable to make social robots that look like imaginary creatures	23	3.304	1.185	80.0	0.126
It is ethically acceptable to make social robots that look like animals	23	3.174	1.193	92.5	0.221

Note. Asterisks indicate that the median is different from 3.

Table 5 Mean scores, with standard deviations, and results of the Wilcoxon signed-rank test for the post-video questionnaire.

Statement	N	Mean	SD	V	p-value
I found the NAO robot complex to use in the ASSISTANT modality	23	2.435	1.161	28.00	0.035*
I found the NAO robot complex to use in the PUPPET modality	23	2.522	0.994	24.00	0.034*
The robot responds quickly enough in the ASSISTANT mode	23	2.957	1.022	50.00	0.894
The robot responds quickly enough in the PUPPET mode	23	3.435	0.788	67.00	0.021*
The actions performed by the robot in the ASSISTANT mode are appropriate with respect to the predetermined objectives	23	3.478	1.082	104.00	0.056
The actions performed by the robot in the PUPPET mode are appropriate with respect to the predetermined objectives	23	3.696	0.635	140.50	<0.001*
The modes of interaction in the ASSISTANT mode are sufficiently rich in relation to the needs	23	3.391	0.839	73.00	0.042*
The modes of interaction in the PUPPET mode are sufficiently rich in relation to the needs	23	3.478	0.790	112.00	0.013*
I would like to use the NAO robot in ASSISTANT mode during medical examinations	23	3.435	1.237	77.00	0.120
I would like to use the NAO robot in PUPPET mode during medical examinations	23	3.652	1.071	90.00	0.017*
It seems to me that the NAO robot in the ASSISTANT mode interferes with the way I prefer to organize my work	23	2.783	1.242	41.00	0.477
It seems to me that the NAO robot in the PUPPET mode interferes with the way I prefer to organize my work	23	2.783	1.204	47.50	0.481
I felt comfortable using the NAO Robot in ASSISTANT mode	23	3.435	0.992	81.50	0.061
I felt comfortable using the NAO Robot in PUPPET mode	23	3.435	0.945	93.00	0.049*
I think that most people can easily learn to use the NAO robot in ASSISTANT mode	23	4.000	0.853	165.00	<0.001*
I think that most people can easily learn to use the NAO robot in PUPPET mode	23	3.739	0.752	129.00	<0.001*
I think the use of the NAO robot in ASSISTANT mode during clinical practice can increase efficiency	23	3.261	0.964	62.50	0.223

Continued on next page

Table 5 – continued from previous page

Statement	N	Mean	SD	V	p-value
I think the use of the NAO robot in ASSISTANT mode during clinical practice can make the work easier	23	3.261	0.915	72.00	0.198
I think the use of the NAO robot in ASSISTANT mode during clinical practice can speed up certain procedures	23	3.348	0.714	55.00	0.037*
I think the use of the NAO robot in ASSISTANT mode may be useful only with certain types of patients	23	3.609	0.988	103.50	0.011*
I think the use of the NAO robot in ASSISTANT mode may frighten the patients	23	2.783	0.902	48.00	0.263
I think the use of the NAO robot in ASSISTANT mode may reassure the patients	23	3.522	1.039	96.00	0.036*
I think the use of the NAO robot in PUPPET mode during clinical practice can increase efficiency	23	3.348	0.885	78.50	0.084
I think the use of the NAO robot in PUPPET mode during clinical practice can make the work easier	23	3.478	0.947	74.50	0.038*
I think the use of the NAO robot in PUPPET mode during clinical practice can speed up certain procedures	23	3.261	0.964	63.50	0.197
I think the use of the NAO robot in PUPPET mode may be useful only with certain types of patients	23	3.435	0.945	64.50	0.042*
I think the use of the NAO robot in PUPPET mode may frighten the patients	23	2.609	1.076	29.00	0.135
I think the use of the NAO robot in PUPPET mode may reassure the patients	23	3.609	1.158	160.00	0.033*
I think I need the support of someone who is already able to use the NAO robot in ASSISTANT mode	23	2.522	1.082	48.00	0.047*
I think I need the support of someone who is already able to use the NAO robot in PUPPET mode	23	3.217	1.085	75.00	0.387
I can understand what to do based solely on the information provided by the system in the ASSISTANT mode	23	2.957	0.976	36.00	0.837
I can understand what to do based solely on the information provided by the system in the PUPPET mode	23	3.174	0.937	42.50	0.402
Using the NAO robot in ASSISTANT mode is frustrating	23	2.217	1.043	16.50	0.004*

Using the NAO robot in PUPPET mode is frustrating	23	2.087	0.900	5.00	<0.001*

* Asterisks indicate that the median is different from 3.
